# PERK participates in cardiac valve development via fatty acid oxidation and endocardial-mesenchymal transformation

**DOI:** 10.1038/s41598-020-77199-4

**Published:** 2020-11-18

**Authors:** Takashi Shimizu, Kazuaki Maruyama, Takeshi Kawamura, Yoshihiro Urade, Youichiro Wada

**Affiliations:** 1 Isotope Science Center, The University of Tokyo, Tokyo, 113-0032 Japan; 2 Department of Cardiovascular Medicine, The University of Tokyo, Graduate School of Medicine, Tokyo, 113-8655 Japan

**Keywords:** Developmental biology, Disease model

## Abstract

Protein kinase R-like endoplasmic reticulum kinase (PERK) is one of the endoplasmic reticulum (ER) stress sensors. *PERK* loss-of-function mutations are known to cause Wolcott–Rallison syndrome. This disease is characterized by early-onset diabetes mellitus, skeletal dysplasia, and cardiac valve malformation. To understand the role of PERK in valve formation in vivo, we used an endothelial-specific PERK conditional knockout mice as well as in vitro PERK inhibition assays. We used ProteoStat dyes to visualize the accumulation of misfolded proteins in the endocardial cushion and valve mesenchymal cells (VMCs). Then, VMCs were isolated from E12.5 fetal mice, by fluorescence assisted cell sorting. Proteomic analysis of PERK-deleted VMCs identified the suppression of proteins related to fatty acid oxidation (FAO), especially carnitine palmitoyltransferase II (CPT2). CPT2 is a critical regulator of endocardial-mesenchymal transformation (EndoMT); however how TGF-β downstream signaling controls CPT2 expression remains unclear. Here, we showed that PERK inhibition suppressed, not only EndoMT but also CPT2 protein expression in human umbilical vein endothelial cells (HUVECs) under TGF-β1 stimulation. As a result, PERK inhibition suppressed mitochondrial metabolic activity. Taken together, these results demonstrate that PERK signaling is required for cardiac valve formation via FAO and EndoMT.

## Introduction

Cardiac valve malformations are among the most common human congenital anomalies, occurring in about 2% of the total live births^1^. Although some of these malformations have been linked to genetic mutations^2^, downstream signaling pathways of these mutations have not been clarified enough.

Cardiac valve development starts from the embryonic day 9.5 (E9.5) with the formation of endocardial cushions^3^. At this time-point, endocardial-mesenchymal transformation (EndoMT), a process in which the endothelial cells transform into mesenchymal cells^4^, occurs at the sites of the future atrioventricular (AV) and the outflow tract (OFT) valves.

EndoMT could be induced by endoplasmic reticulum (ER) stress^5^. When misfolded proteins accumulate in the ER (causing ER stress), the three major arms of the unfolded protein response (UPR) are activated, including the Protein kinase R-like endoplasmic reticulum kinase (PERK), the activating transcription factor 6 (ATF6), and the inositol-requiring enzyme-1 (IRE1). PERK is involved in the initiation of protein synthesis and the activation of the ubiquitin–proteasome protein degradation pathway. PERK phosphorylates the alpha subunit of eukaryotic translation-initiation factor 2 (eIF2α), leading to the repression of global protein synthesis. On the other hand, the translation of some mRNAs with specific upstream open reading frames (uORFs) within their 5′ untranslated region (5′UTR), such as activating transcription factor 4 (ATF4), is promoted by PERK activation. Of note, ATF4 a downstream gene of SMAD signaling, involved in EndoMT.

*PERK* loss-of-function mutations have been known to cause the Wolcott–Rallison syndrome^10^. This syndrome is characterized by early-onset diabetes mellitus, skeletal dysplasia, and can also feature cardiac valve malformation. Of note, PERK knockout mice show early-onset diabetes mellitus and skeletal dysplasia. However, the impact of PERK in cardiac valve formation is unknown.

We hypothesized that PERK downstream signaling may control the protein expressions of EndoMT related genes in VMCs.

First of all, we tried to unravel the role of UPR, especially PERK, in cardiac valve formation using (or by virtue of) endothelial-specific PERK conditional knockout (PERK KO) mice in vivo. To explore the relationship between UPR, including PERK, and EndoMT in vitro, we further used human umbilical vein endothelial cells (HUVECs) with TGF-β1 treatment. Using fluorescence assisted cell sorting (FACS), valve mesenchymal cells (VMCs) in the outflow tract (OFT) valves were isolated from embryos with or without PERK deletion and applied for LC–MS/MS analysis.

## Results

### Presence of misfolded proteins in the hearts of E12.5 embryos

**Figure 1:**
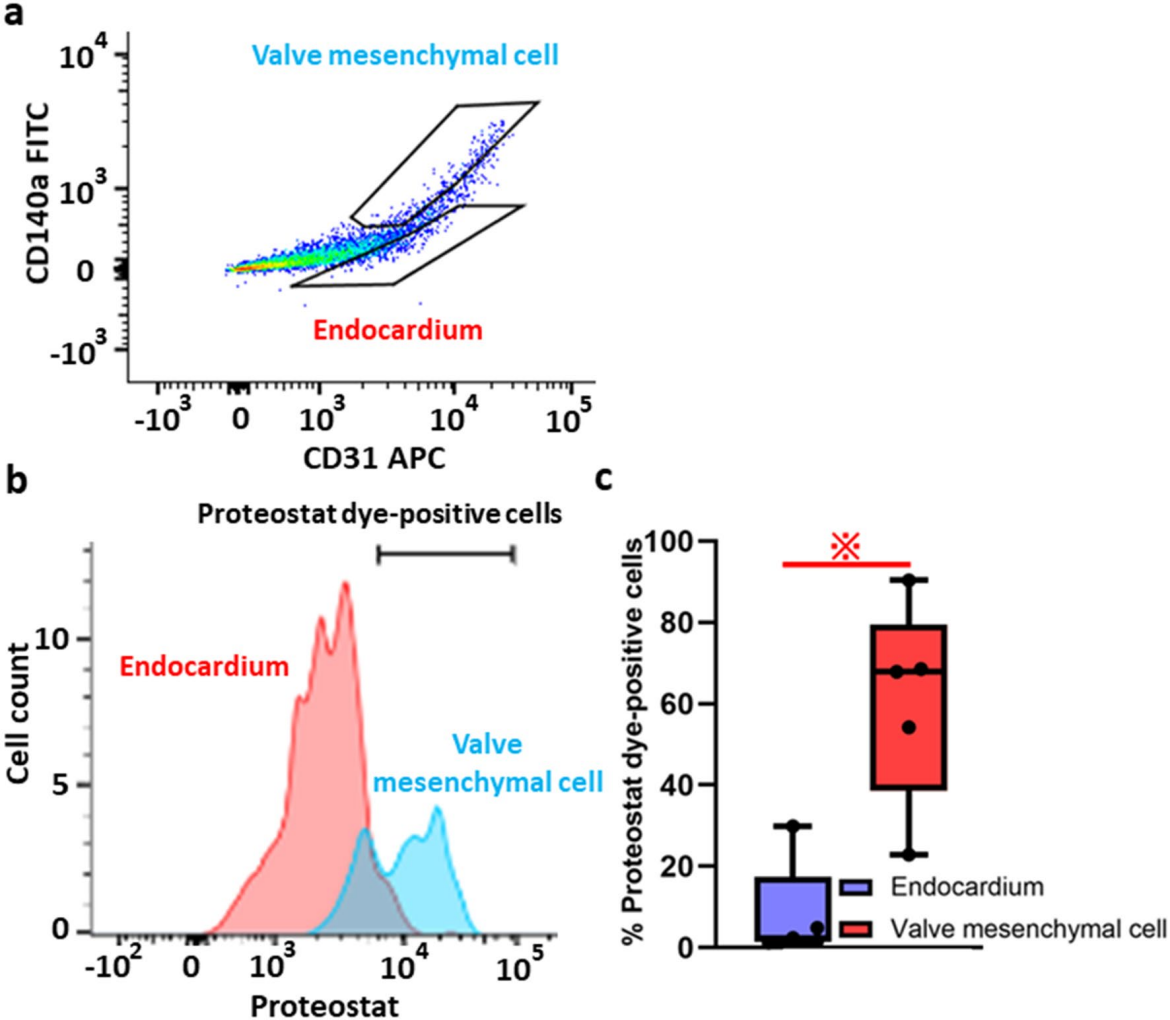
Misfolded proteins accumulate in the endocardial cushions of outflow tract (OFT) valves. (**a**) Flow cytometry plots used to identify endothelial cells on the endocardium (CD31+/CD140a−) and valve mesenchymal cells (VMCs, CD31−/CD140a+). (**b**) Accumulation of misfolded proteins was analyzed and observed on the endocardium (red histogram) and VMCs (blue histogram) using the ProteoStat dye. (**c**) The quantification of the percentage (%) of ProteoStat dye-positive cells is represented (n = 5 per groups). This graph was drawn by Graph Pad Prism8.4.2 (freely available software after purchasing, https://www.graphpad.com/support/faq/prism-842-release-notes/). ^**※**^*p* < 0.05; unpaired *t* test.

To investigate the possible physiological implications of UPR, we tried to detect the accumulation of misfolded proteins in the hearts of C57BL/6 mouse embryos at E12.5, when mesenchymal cell proliferation and condensation in the endocardial cushions are induced by EndoMT^6^. The aggresome-specific ProteoStat dye showed an enhanced fluorescence signal corresponding to the aggregated proteins accumulated in the endocardial cushions (C) of OFT valves, but not in the endocardial cushions of atrioventricular (AV) valves (Supplementary Fig. 1a). To quantify the accumulation of misfolded proteins in endothelial cells and VMCs from the hearts of E12.5 embryos, we used flow cytometry with the ProteoStat dye, anti-CD140a-FITC antibody, and anti-CD31-APC antibody (Fig. 1a,b). The proportion of the ProteoStat-positive cells was higher in VMCs (CD140a/CD31+) than that in endothelial cells (CD31+/CD140a−) (60.8% vs 8.0%, respectively) (Fig. 1c).

### PERK is required for endocardial cushion remodeling during cardiac valve formation

To evaluate the role of PERK in heart development, we used a Tie2-Cre allele, inducing the expression of Cre recombinase in endothelial cells. We inter-crossed male mice homozygous for floxed PERK carrying Tie2-Cre (PERK knockout, KO) with female mice homozygous for floxed PERK (wild type, WT), and produced endothelial-specific PERK KO mice.

**Figure 2:**
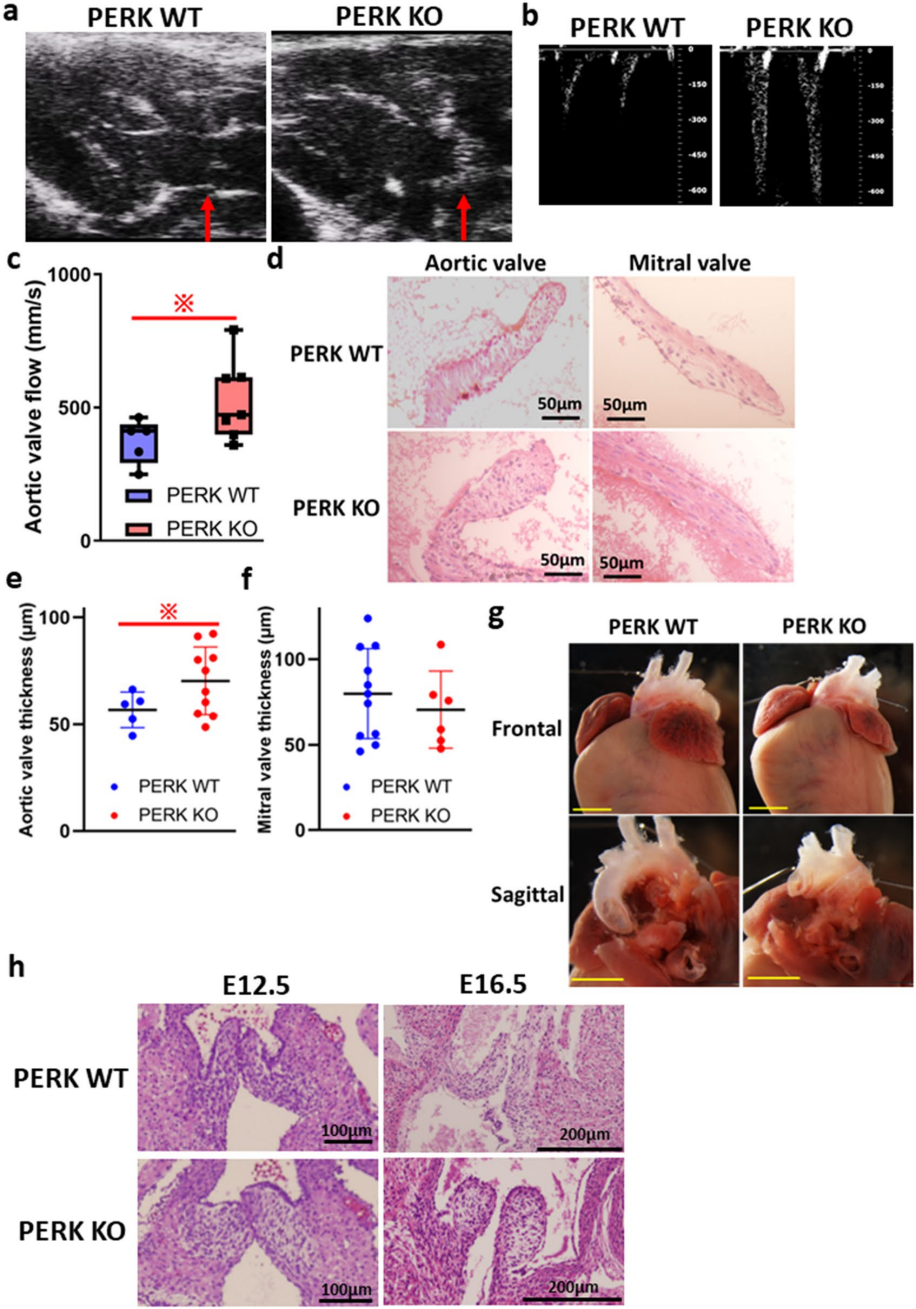
Endocardial loss of PERK results in cardiac valve malformation. (**a**) Aortic valves’ echocardiographic images in adult homozygous floxed PERK mice (PERK wild type, WT; n = 5) or homozygous floxed PERK mice with Tie2-Cre (PERK knockout, KO; n = 7) mice. (**b**) Representative aortic valve (AoV) blood flow velocities in these mice. (**c**) Quantification of the AoV blood flow. (**d**) Hematoxylin and eosin (H&E) staining of the aortic and mitral valves in PERK WT and PERK KO mice. Hearts from adult (8-to-12 weeks old) mice are shown. Scale bar represents 50 μm. (**e**) Quantification of the aortic valve leaflet thickness—referent to (**a**)—(PERK WT; n = 5 and PERK KO; n = 10). Statistical analysis was performed using the unpaired t test. (**f**) Quantification of the mitral valve leaflet thickness—referent to (**a**)—(PERK WT; n = 11 and PERK KO; n = 5). Statistical analysis was performed using the unpaired *t* test. (**g**) Frontal and sagittal images of hearts from PERK WT and PERK KO mice. Scale bar represents 2 mm. (**h**) H&E staining of the aortic valves in PERK WT and PERK KO embryos at E12.5 and E16.5. (**c**,**e**,**f**) were drawn by Graph Pad Prism8.4.2. (freely available software after purchasing, https://www.graphpad.com/support/faq/prism-842-release-notes/ ).

Echocardiography showed that the leaflets of the aortic valve (AoV) were thickened in PERK KO adult mice (Fig. 2a). PERK KO adult mice also exhibited an increased AoV blood flow compared with that in the PERK WT adult mice (529 mm/s vs 375 mm/s, Fig. 2b,c). Moreover, the histological staining revealed that the aortic and mitral valve leaflets were thin in PERK WT adult mice, while the aortic valve leaflets, but not the mitral valve leaflets, were thicker in PERK KO adult mice (Fig. 2d–f). Of note, the OFT alignment was not affected as is shown in Fig. 2g. Taking these together, the onset of functional deficits in the aortic valves of PERK KO mice was confirmed.

Then, to elucidate the pathogenesis, we further analyzed PERK WT and PERK KO embryo hearts (Fig. 2h). The OFT cushions of PERK KO embryos exhibited histologically larger volumes than those in PERK WT embryos at E12.5. Furthermore, they were notably larger than those in control littermates at E16.5 (Fig. 2h).

### PERK deletion suppresses FAO in VMCs

**Figure 3:**
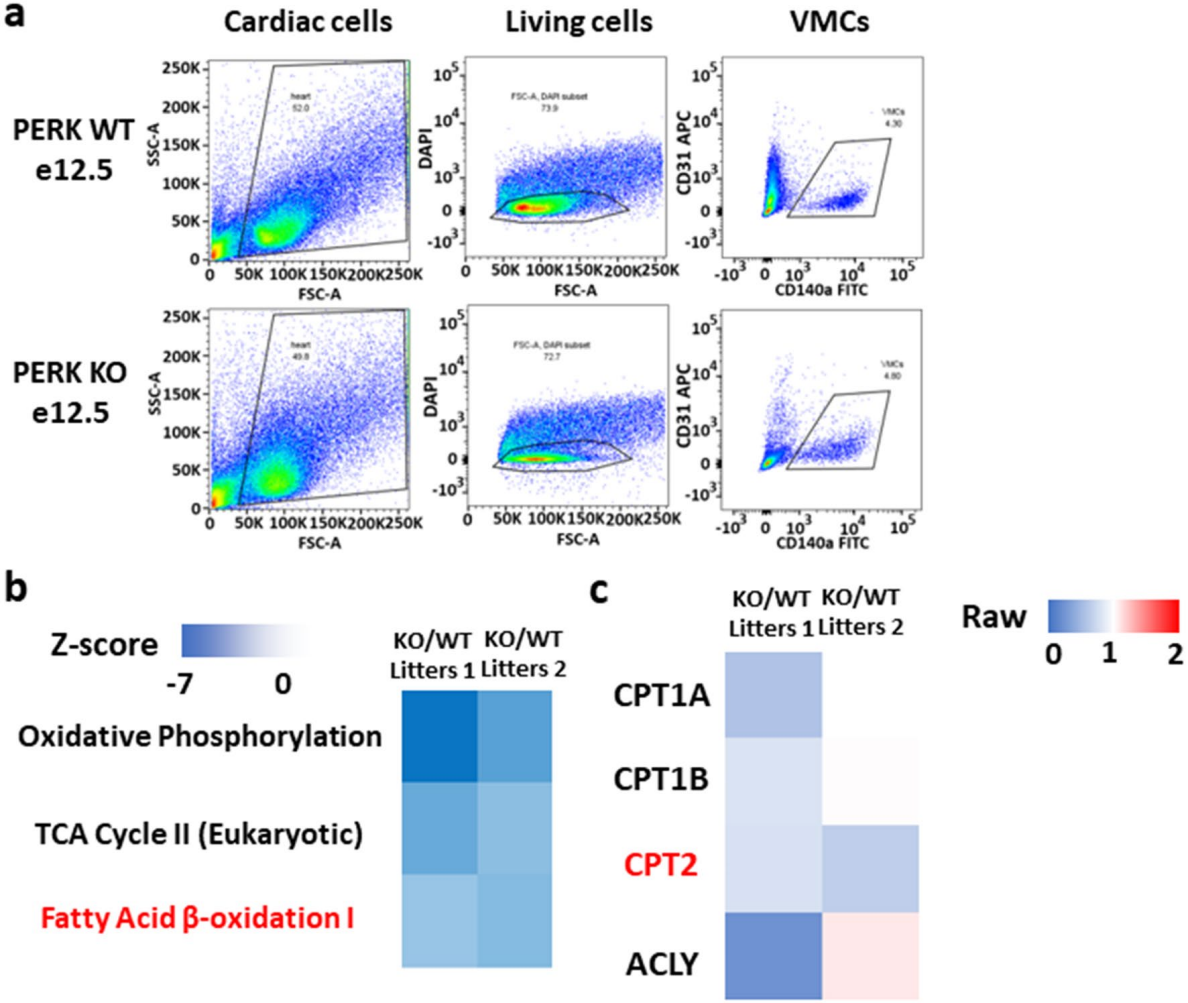
Isolation of valve mesenchymal cells and proteomic analysis. (**a**) Fluorescence-assisted cell sorting (FACS) plots used to identify VMCs from OFT valves (CD31−/CD140a+), isolated from PERK WT and PERK KO E12.5 embryos. (**b**) Signaling pathway analysis of all proteins identified in PERK WT and PERK KO VMCs (two litters). (**c**) Differential Fatty Acid β-oxidation-related protein expression in PERK WT and PERK KO VMCs. The heat-map displays the representative proteins related to FAO (two litters).

To investigate the role of PERK in valve development, we intended to compare proteomic profile between VMCs with or without PERK deletion. Embryos with floxed PERK and heterozygous for Cre were removed and placed into cold, sterile PBS at E12.5 and their hearts were genotyped. Embryos were then separated into Cre-positive (PERK KO) or Cre-negative (PERK WT). Using trypsin, hearts of embryos with similar genotypes were dissociated into single cells; VMCs were isolated using FACS (Fig. 3a). Then, we performed proteomic analysis on these VMCs using LC–MS/MS (two litters). For the canonical pathway analysis, the Ingenuity Pathway Analysis (IPA) software was used. Representative canonical pathways down-regulated in response to PERK inhibition are depicted in Fig. 3b. These include Oxidative Phosphorylation, the TCA Cycle II (Eukaryotic), and Fatty Acid β-oxidation I.

Recently, it has been reported that TGF-β1-induced fatty acid oxidation (FAO) deregulation could trigger EndoMT via the carnitine palmitoyltransferase (CPT) system, which involves two mitochondrial membrane enzymes, CPT1 and CPT2^7^. This system facilitates the transport of long-chain acylcarnitines, metabolites of long-chain fatty acids (FAs), from the cytosol to the mitochondria. CPT1 and CPT2 are indispensable for FAs β-oxidation and for the tricarboxylic acid (TCA) cycle activity^8^. Endothelial deletion of CPT2 results in a cardiac valve malformation, involving the activation of SMAD signaling^7^.

Proteome analyses suggested that, among the gene products in Fatty Acid β-oxidation I, CPT2 was significantly (or specifically) down-regulated by PERK deletion (Fig. 3c). However, the role of PERK involved in TGF-β signaling-derived control of FAO and TCA cycle activity in endothelial cells remains elusive. Therefore we hypothesized that CPT2 participate in EndoMT, which is regulated by PERK cascade.

### Induction of EndoMT is suppressed by PERK inhibition in HUVECs

Based on a previous study^9^, we found that primary cultures of HUVECs could be stimulated to undergo EndoMT by treating with TGF-β1. HUVECs treated with TGF-β1 underwent a clear morphological transition adopting a mesenchymal-like appearance (Fig. 3a).

**Figure 4:**
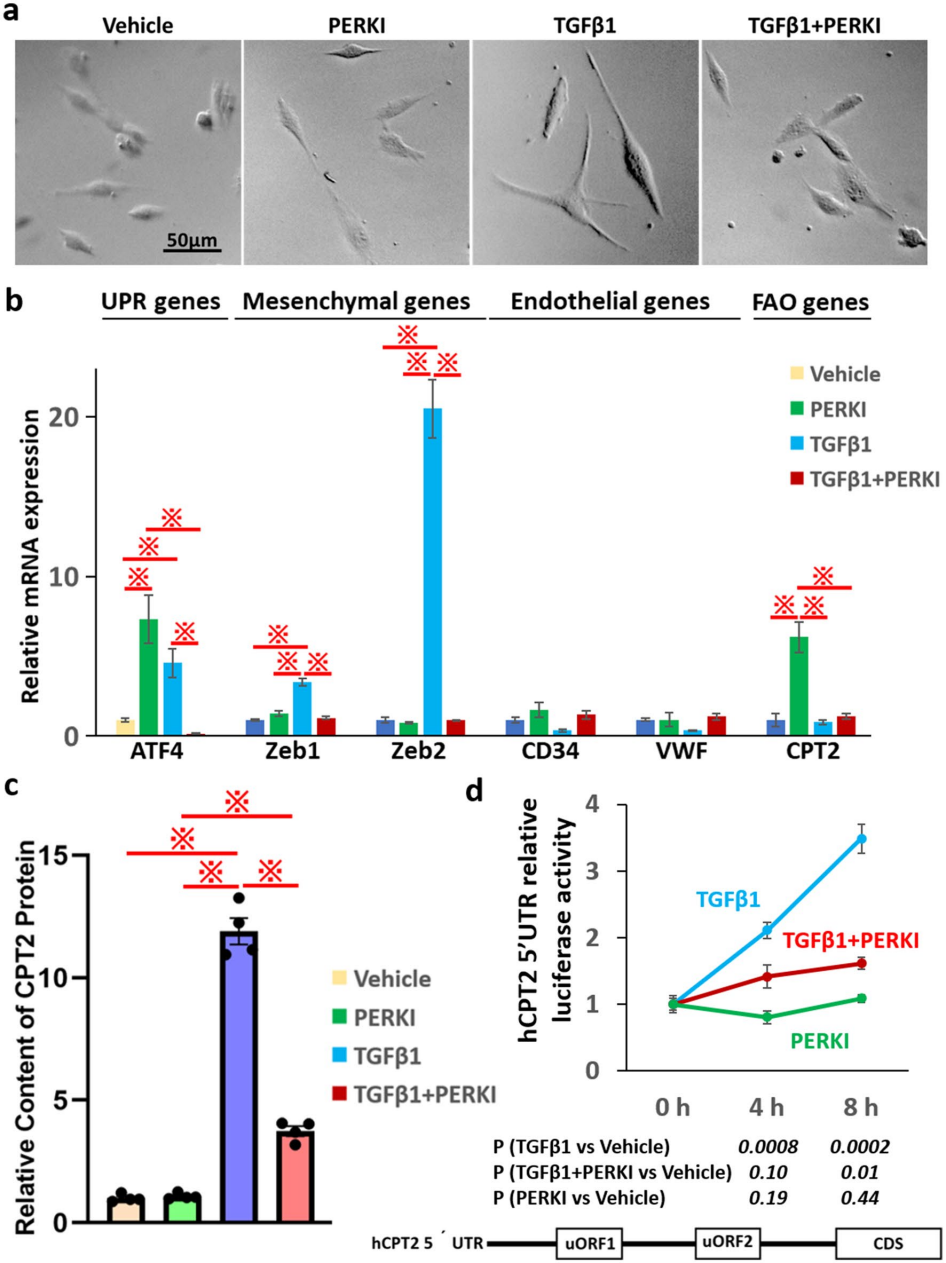
Induction of EndoMT is suppressed by PERK inhibition. (**a**) Morphology of human umbilical vein endothelial cells (HUVECs) treated with vehicle, 1 μM PERK inhibitor (PERKI), TGF-β1, and TGF-β1 + PERKI for 8 h. (**b**) mRNA expression of UPR, EndoMT, and FAO-related genes in HUVECs treated with vehicle (n = 6), PERKI (n = 3), TGF-β1 (n = 3), or TGF-β1 + PERKI (n = 3). Gene expression was detected using qRT-PCR. *β-actin* was used as an internal control. (**c**) mRNA expression of UPR, EndoMT, and the FAO-related genes in HUVECs treated with vehicle, PERKI, TGF-β1, or TGF-β1 + PERKI (n = 4 per group). This graph was drawn by Graph Pad Prism8.4.2 (freely available software after purchasing, https://www.graphpad.com/support/faq/prism-842-release-notes/). (**d**) Schematic representation of the mRNA sequence of human CPT2 (hCPT2). hCPT2 has two uORFs in its 5′UTR. A luciferase-reported assay for the detection of hCPT2 5′UTR in HUVECs treated with vehicle, PERKI, TGF-β1, or TGF-β1 + PERKI for 4 h or 8 h (n = 4 per groups) was performed. P-values were determined using the unpaired t-test. In all bar graphs, the mean ± SEM are shown. In all box-and-whiskers’ plots, mean values are provided. ^**※**^*p* < 0.05; one-way ANOVA with Bonferroni post hoc analysis.

Next, we checked whether HUVECs’ EndoMT could be affected by the treatment with GSK2606414 (PERKI), a selective PERK inhibitor (preventing ER stress-induced PERK auto-phosphorylation). HUVECs treated with PERKI or TGF-β1 + PERKI did not undergo the transition, compared with those treated by vehicle (Fig. 4a).

We further found that, particularly the treatment with TGF-β1 induced the expression of mesenchymal markers, such as Zeb1 and Zeb2, but did not affect that of endothelial markers, such as CD34 and vWF (Fig. 4b). Moreover, the expression of UPR and mesenchymal markers was significantly lower in TGF-β1 + PERKI-treated cells, compared to that in TGF-β1-treated cells (*p* < 0.05). Additionally, the expression of the UPR marker ATF4 was also significantly lower in TGF-β1 + PERKI-treated cells *versus* PERKI-treated cells (*p* < 0.05), while the expression of the mesenchymal markers did not significantly differ between the two groups.

To further explore the consequences of increased ATF4 expression in TGF-β1 and PRRKI-treated cells, we measured the accumulation of misfolded proteins using flow cytometry and the ProteoStat dye (Supplementary Fig. 1b). The proportion of the ProteoStat-positive cells was higher in HUVECs treated with PERKI or TGF-β1 than in cells treated with vehicle (35.9% or 47.9%, respectively vs 5.6%). Moreover, cells treated with TGF-β1 + PERKI showed further higher levels of ProteoStat-positive signals (70%).

As a result, TGF-β1 stimulated EndoMT was cancelled by PERKI in HUVECs, regardless of the accumulation of misfolded proteins.

### PERK inhibition suppress the TGF-β1 responsive translation of CPT2

Since HUVECs was proved to be appropriate model for EndoMT, we further elucidate the molecular effect of PERKI on the mechanism of TGF-β1 responsive CPT2 protein production.

*CPT2* mRNA expression was significantly up-regulated by PERKI only, while that was not changed by TGF-β1 or TGF-β1 (Fig. 4b). To confirm the effect of PERKI from the translational viewpoint, we measured the protein expression of CPT2 using an ELISA kit. The protein expression of CPT2 in TGF-β1-treated cells was the most upregulated, compared with that induced by the other treatments (Fig. 4c). Unexpectedly, positive correlations were not observed between the CPT2 protein and mRNA levels. PERK is known to induce an upstream open reading frame (uORF)-mediated regulation of gene expression. Human CPT2 (hCPT2) mRNA has two uORFs in its 5′UTR (Fig. 4d). Therefore, we hypothesized that PERK promotes CPT2 protein translation. To ascertain this, we performed luciferase reporter assays using HUVECs transfected with a luciferase reporter plasmid containing the 5′ UTR of hCPT2. The relative luciferase activity of each group was normalized to that of the vehicle group (= 100%). The reporter activity was markedly increased upon treatment with TGF-β1 for 4 h (211%; *p* = 0.0008) or 8 h (349%, *p* = 0.0002). This increase was lower in cells treated with TGF-β1 + PERKI for 4 h (142%, non-significant) or 8 h (161%, *p* = 0.01). In contrast, there was no difference comparing cells treated with PERKI for 4 h (80%) or 8 h (109%), and control cells.

The above has led to the conclusion that PERKI suppressed CPT2 protein production under TGF-β1 stimulation conditions.

### PERK inhibition suppresses the TCA cycle activity

CPT2 plays a role in the transport of long-chain acylcarnitines from the cytosol into the mitochondria. Therefore, we speculated TCA cycle activity could be suppressed by PERK inhibition.

**Figure 5:**
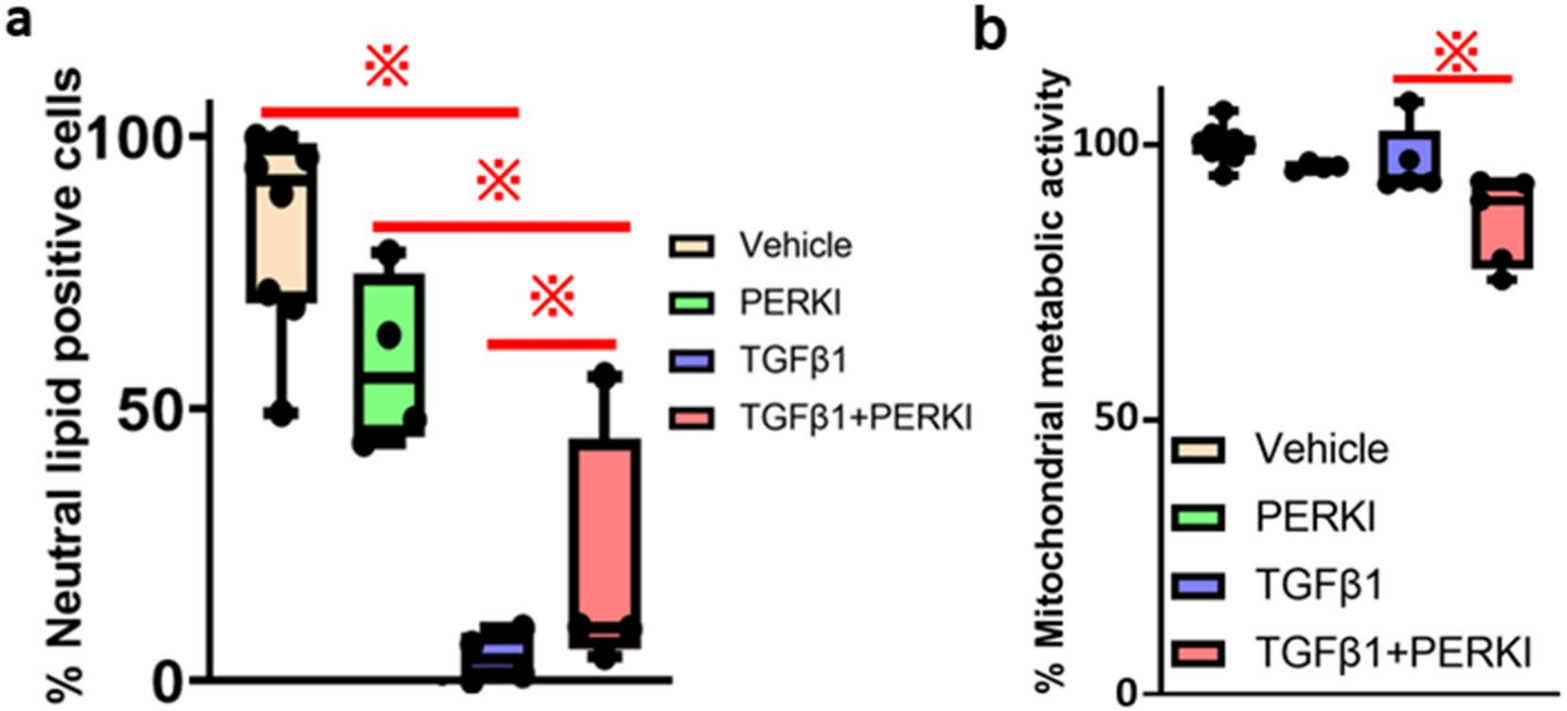
Translation of CPT2 is induced by PERK. (**a**) Flow cytometry-based quantification of BODIPY 493/503 staining in HUVECs treated with vehicle (n = 8), PERKI (n = 4), TGF-β1 (n = 4), or TGF-β1 + PERKI (n = 4). (**b**) Quantification of the mitochondrial metabolic activity of HUVECs treated with vehicle (n = 9), PERKI (n = 4), TGF-β1 (n = 5), or TGF-β1 + PERKI (n = 5), using resazurin. These graphs were drawn by Graph Pad Prism8.4.2 (freely available software after purchasing, https://www.graphpad.com/support/faq/prism-842-release-notes/). In all bar graphs, the mean ± SEM are shown. In all box-and-whiskers’ plots, mean values are provided. ^**※**^*p* < 0.05; one-way ANOVA with Bonferroni post hoc analysis.

First, we examined the effect of PERKI treatment, with or without TGF-β1 on the accumulation of lipid substrates in the cytosol (Fig. 5a). PERKI treatment did not suppress the accumulation of neutral lipid-positive cells (PERKI vs vehicle = 59% vs 84%, respectively). However, under TGF-β1 stimulation, PERKI treatment promoted the accumulation of neutral lipids in the cytosol (TGF-β1 + PERKI vs TGF-β1 = 4% vs 20%, respectively).

Next, we assessed whether PERKI treatment in the presence or absence of TGF-β1 affected the mitochondrial metabolic activity (TCA cycle activity) using resazurin (Fig. 5b). The TCA cycle activity was lower in cells treated with TGF-β1 + PERKI than in cells treated with TGF-β1 alone (TGF-β1 + PERKI vs TGF-β1 = 86% vs 97%, respectively). However, PERKI, alone, did not affect the mitochondrial metabolic activity (PERKI vs vehicle = 96% vs 100%, respectively).

Overall, PERKI inhibited the transport of neutral lipids from the cytosol into the mitochondria in HUVECs under TGF-β1 stimulation, resulting with the suppression of FAO and the TCA cycle during EndoMT and consequently cardiac valve malformation.

## Discussion

In the Wolcott–Rallison syndrome, the mechanism by which PERK affects the cardiac valve formation was thus far unclear. In this study, hearts from endothelial-specific PERK KO embryos revealed significant defects in AoV cushion remodeling (Fig. 2d), but such defects were not seen in the mitral valves (Fig. 2d). This phenotype does not match the traits of the Wolcott–Rallison syndrome, in which mitral valve malformation is observed. The OFT cushion mesenchyme arises not only via EndoMT but also from the neural crest, whereas the AV cushion mesenchyme is solely originated via EndoMT. However, PERK endothelial-specific knockout mice did not show any disorders for the OFT alignment in Fig. 2g. Therefore, endothelial-specific PERK deletion may not affect the migration of cells from the neural crest to the OFT cushion mesenchyme. Another explanation for these differences has to do with the feto-placental circulations. The placenta is the largest fetal organ, and toward the end of pregnancy the umbilical circulation receives at least 40% of the fetal cardiac output^10^. Therefore, there are likely to be close hemodynamic links between the development of the placenta and the fetal heart. Both humans and mice have chorioallantonic placentas, but significant spatio-temporal differences between them are pointed out^11^. In overall inter-species comparison of gestational periods, it is reported that mouse placental development E7.5–18.5 corresponds to human placentae from weeks 4–16. Therefore, the expected different intracardiac distributions of shear stress in human vs murine embryos may justify the differences in valves’ integrity.

In this study, we report that EndoMT is mediated by PERK activity and the subsequent activation of FAO via CPT2 upregulation. These results do not align with a previous study that reported that EndoMT was associated with FAO suppression via CPT2 inhibition or the advancement of TGF-β/SMAD signaling^7^. This discrepancy remains unclarified. *ATF4*, one of the target genes of PERK and TGFβ/SMAD signaling, is known to initiate EndoMT. In Fig. 4b, the expression of ATF4 was increased under TGF-β1 stimulation, while it remained unchanged under TGFβ1 + PERKI stimulation (compared with that in vehicle-treated cells). Therefore, EndoMT may be induced by two different signaling cascades, CPT2-dependent and ATF4-dependent pathways. In future, we will need to examine the relationship between them.

While most of the studies investigating the role of the nongenomic factors during heart development have focused on chromatin accessibility, DNA methylation ^12^, and RNA expression^13^, only a few have addressed this question from a proteomics perspective. This may be due to the fact that proteomics requires larger amounts of samples than those required for other omics analyses. To the best of our knowledge, this is the first LC–MS/MS-based study focusing on cardiac valve development. Of note, without the proteomic analysis, we would not be able to demonstrate that PERK promotes the expression of CPT2. To better understand the coordination between different molecular layers in embryos, there is a need to conduct time-series omics analyses.

## Materials and methods

### Ethics statement

All experiments were approved by the University of Tokyo Ethics Committee for Animal Experiments and strictly adhered to the guidelines for animal experimentation of the University of Tokyo.

### Mice

To generate mice with cardiomyocyte–specific PERK deletion, mice homozygous for the floxed PERK (The Jackson Laboratory, Bar Harbour, ME, USA) were crossed with Tie2-Cre mice. Mice were maintained on a standard rodent chow diet with 12 h light/12 h dark cycles. Mice were bred from the age of 8 weeks. All mice were on a C57BL/6J background and were genotyped by standard PCR-based methods.

### Echocardiographic assessment

Analyses were performed in 8-to-12-week-old mice. Transthoracic echocardiography was performed with a VEVO2100 system (VisualSonics, Toronto, Canada) in non-anesthetized mice.

### Cells

Primary HUVECs were obtained from Lonza (Basel, Switzerland) and were maintained in EGM-2 BulletKit medium (Lonza) at 37 °C in a humidified atmosphere of 5% CO_2_. HUVECs were used between passages 3 and 9.

### Chemicals and reagents

For EndoMT induction experiments, sub-confluent HUVECs were incubated with 10 ng ml^−1^ TGF-β1 (Peprotech, Cranbury, NJ, USA) and/or 1 µM PERK inhibitor (gsk2606414). After 8 h of treatment, *ATF4, Zeb1, CD34, VWF,* and *CPT2* mRNA levels were assessed by real-time quantitative PCR (qPCR). The sequences of the primers used are:

- ATF4 Forward: 5′-tctccagcgacaaggctaa-3′, Reverse: 5′-ccaatctgtcccggagaa-3′;
- Zeb1 Forward: 5′-aggatgacctgccaacagac-3′, Reverse: 5′- atttcttgcccttcctttcc-3′;
- CD34 Forward: 5′-gcgctttgcttgctgagt-3′, Reverse: 5′- gggtagcagtaccgttgttgt-3′;
- VWF Forward: 5′-gaaatgtgtcaggagcgatg-3′, Reverse: 5′-atccaggagctgtccctca-3′;
- CPT2 Forward: 5′-ggtgctctgaggcctttgt-3′, Reverse: 5′-cctggcttgctgtgtaacg-3′.

CPT2 protein expressions in these cells were quantified with a Human Carnitine Palmitoyltransferase 2, Mitochondrial (CPT2) ELISA Kit (MyBioSource, San Diego, CA, USA). Protein was extracted from cells and then subjected to ELISA according to the manufacturer's instructions. All CPT2 protein levels were normalized to the total protein levels.

### Luciferase reporter assay

Plasmids were constructed cloning 429 base pairs of the *hCPT2* 5′UTR into the HindIII/ApaI sites of a pGL4.13[luc2/SV40] vector (Promega, Madison, WI, USA), downstream of the sequence encoding for the luc2 reporter gene. Using Lipofectamine 3000, plasmids (150 ng) were transfected into HEK293T cells in a 96-well plate and the luciferase activity measured 24 h later using the One-Glo luciferase assay (Promega).

### Cell isolation from the embryos

Endothelial cells and VMCs from the E12.5 embryos were isolated by the fluorescence-activated cell sorting (FACS) using a BD FACSAria (BD Biosciences, San Jose, CA, USA). Homozygous floxed PERK Tie2-Cre male mice were crossed to homozygous floxed PERK females. Hearts of the E12.5 embryos were micro-dissected and placed into cold, sterile PBS. Then, they were pooled into 300 μl Trypsin–EDTA (0.25%; Gibco) and incubated at 37 °C for 30 min. Subsequently, 300 μl cold, sterile 5% glucose was added. The samples were then filtered through a 70-μm cell strainer. Isolated cells were kept on ice until they were used for FACS. The dead cells and debris were excluded based on forward scatter/side scatter (FSC/SSC) and DAPI signals (1:1000 dilution). To identify endothelial cells and VMCs, the cells were stained with the following antibodies for 3 h at 4 °C: APC anti-mouse CD31 antibody (1:100 dilution, Invitrogen, Carlsbad, CA, USA) and FITC anti-mouse CD140a (PDGFRα) antibody (1:100 dilution, Invitrogen).

### Histological analyses

For histological analyses, mouse embryos were fixed in 4% paraformaldehyde (PFA), and embedded in paraffin, as described elsewhere^6^. Sections (4-μm thick) were used for immunohistochemistry, or hematoxylin and eosin (H&E) staining. Image acquisition was performed under a fluorescence microscope (FSX100, Olympus Life Science, Waltham, MA, USA), or an optical or phase-contrast microscope, respectively.

Protein aggregation was assessed in hearts of E12.5 embryos, using the ProteoStat protein aggregation assay kits (Enzo Life Sciences, Farmingdale, NY, USA). ProteoStat emits fluorescence when it binds to the tertiary structure of aggregated proteins. It has been validated to specifically detect protein aggregates and aggresome-like inclusion bodies in cells. To label the protein aggregates, tissue sections were incubated with the ProteoStat dye for 30 min at room temperature (about 20 °C). All sections were counterstained with nuclear Hoechst (1:1000). Image acquisition was performed under a fluorescence microscope (FSX100, Olympus Life Science).

### Protein aggregation assays

Cytoplasmic protein aggregation or inclusion body formation in HUVECs or isolated endothelial cells and VMCs from E12.5 embryos were analyzed as per previously described methods^14,15^. Briefly, these cells were fixed with 4% paraformaldehyde for 15 min. At the end of fixation, cells were washed with PBS and incubated at room temperature for 15 min with a permeabilization buffer. Cells were then incubated with the ProteoStat dye^16^, Hoechst, APC-anti-CD31 antibody, and FITC-anti-CD140a antibody for 30 min. Fluorescence was measured using flow cytometry.

### Neutral lipids’ staining

HUVECs were stained with 2 μM boron-dipyrromethene (BODIPY) 493/503^17^ (D3922; Thermo Fisher) for 15 min. Then, cells were washed with PBS and analysed by flow cytometry.

### Resazurin assay

The resazurin assay is widely used to measure mitochondrial metabolic activity. HUVECs were seeded (100-μl volume per well) in 96-well plates and grown for a few days. Cells were then exposed to PERKI, TGF-β1, or TGF-β1 + PERKI. After 6 h, resazurin ready-to-use solution (TCI) was added—a volume equal to 10% of the cell culture media volume—and incubated for 2 h to 3 h at 37 °C. The fluorescence intensity was measured using 544 nm excitation and 590 nm emission wavelengths.

### Mass spectrometry analysis

Protein trypsin digestion was performed as per a previously described method^18^. Tandem mass spectrometry was performed using an LTQ Orbitrap ELITE ETD mass spectrometer (Thermo Fisher Scientific, Waltham, MA, USA). The methods used for liquid chromatography-tandem mass spectrometry (LC–MS/MS) were slightly modified from those described previously^18^. The mass spectrometer was operated in a data-dependent acquisition mode, in which MS acquisition with a mass range of 400–1000 m/z was automatically switched to the MS/MS acquisition under the control of the Xcalibur software. The top four precursor ions in the MS scan were selected by Orbitrap, with the resolution R = 240,000; those in subsequent MS/MS scans were selected in an ion-trap via the automated gain control (AGC) mode, where AGC values were 1 × 10^6^ and 1 × 10^4^ for full MS and MS/MS, respectively. For fragmentation, the electron transfer dissociation (ETD) was used. MS/MS spectra were then searched against the SwissProt mouse database (version 2019-07) using Mascot (Matrix Science, London, UK). The search results for protein identification were validated using the post-analysis software package Scaffold 4.9.0 (Proteome Software Inc., Portland, OR, USA). When the peptide threshold % false discovery rate (%FDR) was < 1%, peptide identification was accepted. Protein identification was accepted when at least two unique peptides were identified, and the significance threshold was %FDR < 1.

### Bioinformatics

Functional and canonical pathways’ analyses were performed using the Ingenuity Pathway Analysis software (IPA) (Ingenuity Systems, https://www.ingenuity.com) and the label-free quantitative LC–MS/MS data collected from VMCs with or without PERK deletion, as described previously^19^.

All proteins from the dataset were considered for the analyses. Fold changes (FCs) of protein expression were computed for the entire expression dataset and uploaded for the IPA core analysis. The dynamic canonical pathways generated by IPA have been curated and hand-drawn from specific journal articles, review articles, textbooks, and the Kyoto Encyclopaedia of Genes and Genomes (KEGG). The significance of the association between the dataset and the canonical pathway was measured. The Activation z-score was used to infer the activation states of the implicated biological functions.

### Statistical analysis

The Student's *t *test or one-way ANOVA with a Bonferroni post hoc analysis were used for comparisons between 2, or more that 2 groups, respectively. The R software was used for the statistical analysis. Heat-map clustering was also performed with the R software.

## Supplementary information

Supplementary Figure 1.

## Data Availability

All data generated or analyzed in this study are included in this article and its supplementary information files. All of the MS proteomics data have been deposited in JPST000945 (Japan Proteome Standard Repository/Database), or PXD0 21044 (ProteomeXchange Datasets).

## References

[1] Hoffman JI, Kaplan S (2002). The incidence of congenital heart disease. J. Am. Coll. Cardiol..

[2] Wirrig EE, Yutzey KE (2014). Conserved transcriptional regulatory mechanisms in aortic valve development and disease. Arterioscler. Thromb. Vasc. Biol..

[3] Gitler AD, Lu MM, Jiang YQ, Epstein JA, Gruber PJ (2003). Molecular markers of cardiac endocardial cushion development. Dev. Dyn..

[4] Markwald RR, Fitzharris TP, Manasek FJ (1977). Structural development of endocardial cushions. Am. J. Anat..

[5] Ying R (2016). Hydrogen sulfide suppresses endoplasmic reticulum stress-induced endothelial-to-mesenchymal transition through Src pathway. Life Sci..

[6] Goddard LM (2017). Hemodynamic forces sculpt developing heart valves through a KLF2-WNT9B paracrine signaling axis. Dev. Cell.

[7] Xiong J (2018). A metabolic basis for endothelial-to-mesenchymal transition. Mol. Cell.

[8] Nomura M (2016). Fatty acid oxidation in macrophage polarization. Nat. Immunol..

[9] Chen X (2015). Protective effect of spironolactone on endothelial-to-mesenchymal transition in HUVECs via notch pathway. Cell Physiol. Biochem..

[10] Burton GJ, Jauniaux E (2018). Development of the human placenta and fetal heart: synergic or independent?. Front. Physiol..

[11] Soncin F (2018). Comparative analysis of mouse and human placentae across gestation reveals species-specific regulators of placental development. Development.

[12] Gilsbach R (2014). Dynamic DNA methylation orchestrates cardiomyocyte development, maturation and disease. Nat. Commun..

[13] Su T (2018). Single-cell analysis of early progenitor cells that build coronary arteries. Nature.

[14] Zhao Z, Cao L, Reece EA (2017). Formation of neurodegenerative aggresome and death-inducing signaling complex in maternal diabetes-induced neural tube defects. Proc. Natl. Acad. Sci. U.S.A..

[15] Harischandra DS, Jin H, Anantharam V, Kanthasamy A, Kanthasamy AG (2015). alpha-Synuclein protects against manganese neurotoxic insult during the early stages of exposure in a dopaminergic cell model of Parkinson's disease. Toxicol. Sci..

[16] Leeman DS, Ruetz T, Webb AE, McKay A, Pollina EA, Dulken BW, Zhao X, Yeo RW, Ho TT, Mahmoudi S, Devarajan K, Passegué E, Rando TA, Frydman J, Brunet A (2018). Lysosome activation clears aggregates and enhances quiescent neural stem cell activation during aging. Science.

[17] Nomura M (2019). Macrophage fatty acid oxidation inhibits atherosclerosis progression. J. Mol. Cell. Cardiol..

[18] Daigo K, Kawamura T, Matsubara K, Jiang S, Ohashi R, Sudou Y, Kodama T, Naito M, Inoue K, Hamakubo T (2012). The proteomic profile of circulating pentraxin 3 (PTX3) complex in sepsis demonstrates the interaction with azurocidin 1 and other components of neutrophil extracellular traps. Mol. Cell. Proteomics.

[19] Yao C (2015). Protein expression by human pulmonary artery smooth muscle cells containing a BMPR2 mutation and the action of ET-1 as determined by proteomic mass spectrometry. Int. J. Mass Spectrom..

