# Author Correction: PERK participates in cardiac valve development via fatty acid oxidation and endocardial-mesenchymal transformation

**DOI:** 10.1038/s41598-021-85025-8

**Published:** 2021-02-26

**Authors:** Takashi Shimizu, Kazuaki Maruyama, Takeshi Kawamura, Yoshihiro Urade, Youichiro Wada

**Affiliations:** 1 Isotope Science Center, The University of Tokyo, Tokyo, 113-0032 Japan; 2 Department of Cardiovascular Medicine, The University of Tokyo, Graduate School of Medicine, Tokyo, 113-8655 Japan

Correction to: *Scientific Reports* 10.1038/s41598-020-77199-4, published online 18 November 2020

In this Article, the legend of Figure 4 is incorrect:

“Induction of EndoMT is suppressed by PERK inhibition. (a) Morphology of human umbilical vein endothelial cells (HUVECs) treated with vehicle, 1 μM PERK inhibitor (PERKI), TGF-β1, and TGF-β1 + PERKI for 8 h. (b) mRNA expression of UPR, EndoMT, and FAO-related genes in HUVECs treated with vehicle (n = 6), PERKI (n = 3), TGF-β1 (n = 3), or TGF-β1 + PERKI (n = 3). Gene expression was detected using qRT-PCR. β-actin was used as an internal control. (c) mRNA expression of UPR, EndoMT, and the FAO-related genes in HUVECs treated with vehicle, PERKI, TGF-β1, or TGF-β1 + PERKI (n = 4 per group). This graph was drawn by Graph Pad Prism8.4.2. (freely available software after purchasing, https://www.graphpad.com/support/faq/prism-842-release-notes/). (d) Schematic representation of the mRNA sequence of human CPT2 (hCPT2). hCPT2 has two uORFs in its 5’UTR. A luciferase-reported assay for the detection of hCPT2 5′UTR in HUVECs treated with vehicle, PERKI, TGF-β1, or TGF-β1 + PERKI for 4 h or 8 h (n = 4 per groups) was performed. P-values were determined using the unpaired t-test. In all bar graphs, the mean ± SEM are shown. In all box-and-whiskers’ plots, mean values are provided. ※p < 0.05; one-way ANOVA with Bonferroni post hoc analysis.”

should read:

“Induction of EndoMT is suppressed by PERK inhibition. (a) Morphology of human umbilical vein endothelial cells (HUVECs) treated with vehicle, 1 μM PERK inhibitor (PERKI), TGF-β1, and TGF-β1 + PERKI for 8 h. (b) mRNA expression of UPR, EndoMT, and FAO-related genes in HUVECs treated with vehicle (n = 6), PERKI (n = 3), TGF-β1 (n = 3), or TGF-β1 + PERKI (n = 3). Gene expression was detected using qRT-PCR. β-actin was used as an internal control. (c) Protein expression of human CPT2 (hCPT2) in HUVECs treated with vehicle, PERKI, TGF-β1, or TGF-β1 + PERKI (n = 4 per group). This graph was drawn by Graph Pad Prism8.4.2. (freely available software after purchasing, https://www.graphpad.com/support/faq/prism-842-release-notes/). (d) Schematic representation of the mRNA sequence of hCPT2. hCPT2 has two uORFs in its 5’UTR. A luciferase-reported assay for the detection of hCPT2 5′UTR in HUVECs treated with vehicle, PERKI, TGF-β1, or TGF- β1 + PERKI for 4 h or 8 h (n = 4 per groups) was performed. P-values were determined using the unpaired t-test. In all bar graphs, the mean ± SEM are shown. In all box and whiskers’ plots, mean values are provided. p < 0.05; one way ANOVA with Bonferroni post hoc analysis.”

Additionally, the legend of Figure 5 is incorrect:

“Translation of CPT2 is induced by PERK. (a) Flow cytometry-based quantification of BODIPY 493/503 staining in HUVECs treated with vehicle (n = 8), PERKI (n = 4), TGF-β1 (n = 4), or TGF-β1 + PERKI (n = 4). (b) Quantification of the mitochondrial metabolic activity of HUVECs treated with vehicle (n = 9), PERKI (n = 4), TGF-β1 (n = 5), or TGF-β1 + PERKI (n = 5), using resazurin. These graphs were drawn by Graph Pad Prism8.4.2. (freely available software after purchasing, https://www.graphpad.com/support/faq/prism-842-release-notes/). In all bar graphs, the mean ± SEM are shown. In all box-and-whiskers’ plots, mean values are provided. ※p < 0.05; one-way ANOVA with Bonferroni post hoc analysis.’’

should read:

“Transport of neutral lipids from the cytosol into the mitochondria is induced by PERK. (a) Flow cytometry-based quantification of BODIPY 493/503 staining in HUVECs treated with vehicle (n = 8), PERKI (n = 4), TGF-β1 (n = 4), or TGF-β1 + PERKI (n = 4). (b) Quantification of the mitochondrial metabolic activity of HUVECs treated with vehicle (n = 9), PERKI (n = 4), TGF-β1 (n = 5), or TGF-β1 + PERKI (n = 5), using resazurin. These graphs were drawn by Graph Pad Prism8.4.2. (freely available software after purchasing, https://www.graphpad.com/support/faq/prism842-release-notes/). In all bar graphs, the mean ± SEM are shown. In all box-and-whiskers’ plots, mean values are provided. p < 0.05; one-way ANOVA with Bonferroni post hoc analysis.”

