# Supplementary material for: PERK participates in cardiac valve development via fatty acid oxidation and endocardial-mesenchymal transformation
Source: Sci Rep. 2020 Nov 18;10:20094. doi: 10.1038/s41598-020-77199-4 (PMC7674462; doi:10.1038/s41598-020-77199-4)

**PERK participates in cardiac valve development via fatty acid oxidation and endocardial-mesenchymal transformation**

Takashi Shimizu¹^,^²^,^*, Kazuaki Maruyama^1^, Takeshi Kawamura¹, Yoshihiro Urade¹, Youichiro Wada¹

**Supplementary Figure 1. Accumulation of misfolded proteins in HUVECs under TGF-β1 stimulation**

1. Misfolded proteins (red) in E12.5 developing heart valves, such as OFT and atrioventricular (AV) valves, were detected by immunostaining with the ProteoStat dye. Hoechst (blue) denotes the nuclei. Arrows show specific signals.
2. Aggregation of misfolded proteins was analyzed in HUVECs treated with vehicle (n=8), PERKI (n=4), TGF-β1 (n=4) and TGF-β1 + PERKI (n=4), using the ProteoStat dye.

In all bar graphs, the mean ± SEM are shown. In all box-and-whiskers’ plots, mean values are provided. ※ p < 0.05; one-way ANOVA with Bonferroni post hoc analysis.


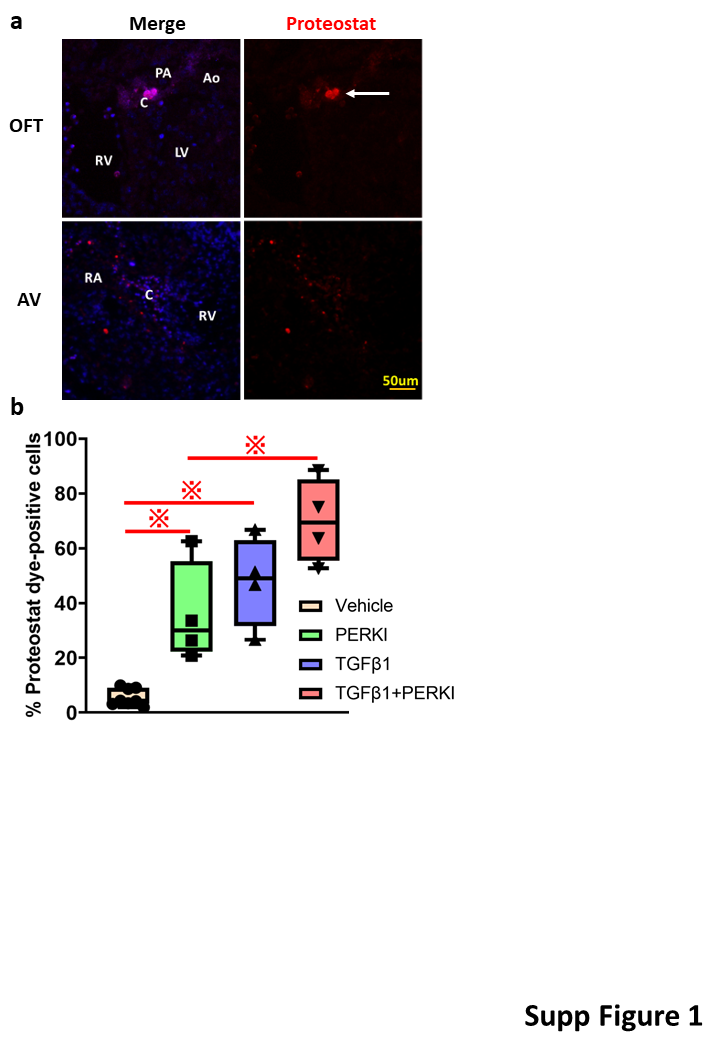

Supplement: Supplementary file 1 — Supplementary Figure 1. [file 41598_2020_77199_MOESM1_ESM.docx]
